# The rapid assessment of avoidable blindness (RAAB) survey methodology

**Published:** 2023-01-30

**Authors:** Ian McCormick

**Affiliations:** Research Fellow: International Centre for eye Health, London School of Hygiene and Tropical Medicine, London, UK.

## Abstract

Rapid assessment of avoidable blindness (RAAB) is a population-based survey methodology that is designed to provide a simple and affordable – yet reliable – estimate of the prevalence and causes of vision impairment and blindness among people aged 50 years and older in a defined population.^1^

The locally relevant data that RAAB surveys provide are used by governments and non-governmental eye health service providers to support evidence-based eye care planning, eye service monitoring and evaluation. RAAB is therefore an important tool in achieving the global eye health priorities set out by the World Health Organization's World Report on Vision and the Lancet Global Health Commission on Global Eye Health.^2^^,^^3^

RAAB surveys provide the majority of the data used to estimate the global and regional prevalence and causes of vision impairment, as well as data which are vital for tracking progress towards eye health within universal health coverage, such as effective cataract surgical coverage and effective refractive error coverage.^4^^,^^5^

The RAAB repository (www.raab.world) collates RAAB survey results and datasets and makes them available for secondary analyses; data from 118 of the 330 RAABs carried out since 2000 have has been made available for this purpose. We encourage more RAAB survey principal investigators and data owners to share their data via the repository, so that the global eye health community collectively can have a more comprehensive and powerful evidence base for research and advocacy.

**Figure:**
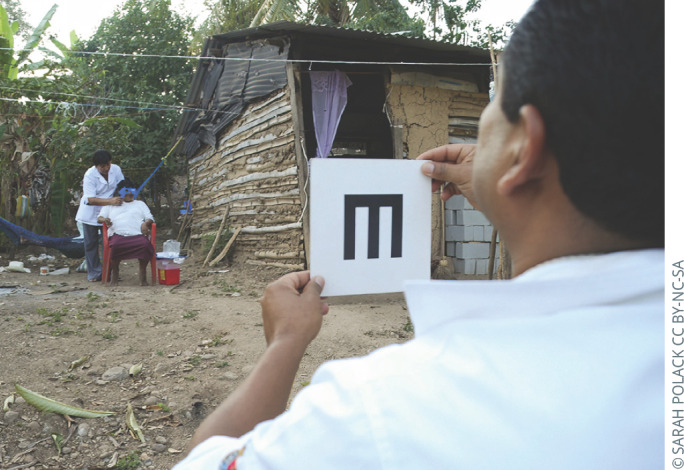
Visual acuity testing during a RAAB survey. mexico
